# Harnessing plant growth-promoting rhizobacteria, *Bacillus subtilis* and *B. aryabhattai* to combat salt stress in rice: a study on the regulation of antioxidant defense, ion homeostasis, and photosynthetic parameters

**DOI:** 10.3389/fpls.2024.1419764

**Published:** 2024-06-13

**Authors:** Ayesha Siddika, Alfi Anjum Rashid, Shakila Nargis Khan, Amena Khatun, Muhammad Manjurul Karim, P.V. Vara Prasad, Mirza Hasanuzzaman

**Affiliations:** ^1^ Department of Agronomy, Faculty of Agriculture, Sher-e-Bangla Agricultural University, Dhaka, Bangladesh; ^2^ Department of Microbiology, University of Dhaka, Dhaka, Bangladesh; ^3^ Department of Agriculture, Noakhali Science and Technology University, Noakhali, Bangladesh; ^4^ Department of Agronomy, Kansas State University, Manhattan, KS, United States

**Keywords:** abiotic stress, AsA-GSH pathway, auxin, *Bacillus*, ion homeostasis, osmotic stress, stress signaling

## Abstract

**Introduction:**

The ongoing global expansion of salt-affected land is a significant factor, limiting the growth and yield of crops, particularly rice (*Oryza sativa* L). This experiment explores the mitigation of salt-induced damage in rice (cv BRRI dhan100) following the application of plant growth-promoting rhizobacteria (PGPR).

**Methods:**

Rice seedlings, at five- and six-weeks post-transplanting, were subjected to salt stress treatments using 50 and 100 mM NaCl at seven-day intervals. Bacterial cultures consisting of endophytic PGPR (*Bacillus subtilis* and *B. aryabhattai*) and an epiphytic PGPR (*B. aryabhattai*) were administered at three critical stages: transplantation of 42-day-old seedlings, vegetative stage at five weeks post-transplantation, and panicle initiation stage at seven weeks post-transplantation.

**Results:**

Salt stress induced osmotic stress, ionic imbalances, and oxidative damage in rice plants, with consequent negative effects on growth, decrease in photosynthetic efficiency, and changes in hormonal regulation, along with increased methylglyoxal (MG) toxicity. PGPR treatment alleviated salinity effects by improving plant antioxidant defenses, restoring ionic equilibrium, enhancing water balance, increasing nutrient uptake, improving photosynthetic attributes, bolstering hormone synthesis, and enhancing MG detoxification.

**Discussion:**

These findings highlight the potential of PGPR to bolster physiological and biochemical functionality in rice by serving as an effective buffer against salt stress–induced damage. *B. subtilis* showed the greatest benefits, while both the endophytic and epiphytic *B. aryabhattai* had commendable effects in mitigating salt stress–induced damage in rice plants.

## 1 Introduction

The escalation of urbanization and industrialization across the globe has decreased the areas of available fertile agricultural land in conjunction with substantial increases in the global population (Sharma and Kumawat, 2022). This scenario has necessitated urgent improvements in agricultural productivity to meet current and future food demands. However, the intensifying environmental stress arising from global climate change is also adversely affecting crop yield by exacerbating stresses due to various abiotic factors, including salinity, drought, waterlogging, heat stress, cold injury, light stress, UV radiation, toxic metal/metalloid stress, ozone exposure, and even soil nutrient toxicity. Of these abiotic stresses, salinity affected area is showing expansion and is particularly concerning, as it is not only destructive to growing plants, but it also renders vast areas of agricultural lands unfit for crop cultivation (Khasanov et al., 2023).

Soil salinity is characterized by the excessive accumulation of salts, such as sodium (Na^+^), chloride (Cl^−^), potassium (K^+^), and calcium (Ca^2+^), in the soils, with Na^+^ and Cl^−^ as the dominant ion species. Elevated salt ion concentrations in soil disrupt natural soil processes (e.g., soil nutrient imbalance, microbial activity inhibition, reduced water infiltration, soil structure degradation, etc.), ultimately impeding plant growth and productivity (Munns, 2011). Salinity influences every phase of a plant’s life cycle, from germination to yield, by altering morphophysiological and biochemical processes (Roman et al., 2020). In particular, plants growing in saline environments produce high levels of reactive oxygen species (ROS). Plants have their innate ability to prevent the generation of ROS during normal photosynthetic and respiratory metabolism through antioxidant defense systems. However, overly-produced ROS under saline conditions overwhelms the inherent antioxidant defense systems, resulting in oxidative stress in plants (Basit et al., 2023). Salinity, therefore, creates challenges to sustainable agriculture and the production of sufficient food to meet global food requirements and ensure future food and nutritional security.

One strategy for overcoming the deleterious effects of saline soils is to use plant growth-promoting rhizobacteria (PGPR). These microbes have gained attention in recent years for their potential to enhance soil ecosystems and improve crop yields in stressful environments by colonizing the plant root system or rhizosphere and stimulating growth without incurring negative impacts on the surrounding environment. PGPR enhance plant growth either directly or indirectly by fixing atmospheric nitrogen, solubilizing essential nutrient elements (e.g., phosphorus [P], potassium [K], zinc [Zn]); producing phytohormones (e.g., indole-3-acetic acid [IAA]), exopolysaccharides (EPS), siderophores, 1-aminocyclopropane-1-carboxylate deaminase, and antioxidants; suppressing diseases through antibiotic production; bolstering plant resistance to biotic and tolerance to abiotic stresses; and promoting plant-microbe symbiosis (Chakraborty et al., 2021; Dame et al., 2021). The ability of PGPR to alleviate environmental stress effects in plants improves plant growth and stress tolerance; therefore, PGPR can serve as ecological engineers for climate-smart farming.

The PGPR bacterial genera include *Agrobacterium*, *Azospirillum*, *Arthrobacter*, *Azotobacter*, *Rhizobium*, *Bacillus*, *Erwinia*, *Bradyrhizobium*, *Burkholderia*, *Pseudomonas*, *Achromobacter*, *Enterobacter*, *Chromobacterium*, among others, but all induce plant tolerance to salinity and other abiotic stresses to promote overall plant growth under stressful conditions. For instance, *Bacillus* sp. is a notable PGPR that enhances the morphophysiological attributes of plants in ways that aid plant survival under stressful conditions. Applications of *Bacillus* sp. in the soil as well as in plants improve plant growth, enhance water retention, reduce ionic toxicity, suppress membrane damage, and maintain electrical conductivity to mitigate salt-induced damage (Ji et al., 2022; Hasanuzzaman et al., 2022b). Beneficial effects are recognized for both endophytic PGPR, such as *B*. *subtilis* (Woo et al., 2020; Hasanuzzaman et al., 2022b) and *B*. *aryabhattai*, as well as epiphytic PGPR, such as *B*. *aryabhattai* (Sultana et al., 2020, 2021), in promoting plant stress tolerance.

This study aimed to assess the effects of salt stress on rice physiology and growth, with a focus on evaluating the potential of *B*. *subtilis* and *B*. *aryabhattai* to mitigate oxidative damage under salt stress conditions. Rice is a staple food for over half of the world’s population, making it crucial to ensure its resilience to environmental stressors like salinity. However, there is limited research on the specific roles of *Bacillus* species in alleviating oxidative stress in rice plants under salt stress conditions. Sea levels rise as a consequence of climate change causing seawater flooding and making rice cultivation difficult in the coastal areas during dry seasons (January-May) (SRDI, 2010). Therefore, rice cultivation during this period provides additional production to meet the global demand for rice (Jahan et al., 2023). Hence, the aim of the present study was to assess salt stress effects on the physiology and growth of rice. The main goal was to explore the extent of damage inflicted on rice exposed to salinity stress and to determine whether the presence of the endophytic PGPR, *B*. *subtilis* and *B*. *aryabhattai*, and the epiphytic PGPR, *B*. *aryabhattai*, can mitigate oxidative damage in rice under salt stress conditions. The findings will contribute to the broader goal of understanding and enhancing PGPR-mediated salt stress tolerance in rice.

## 2 Materials and methods

### 2.1 Plant materials, growing conditions, experimental treatments, and design

**Figure 1:**
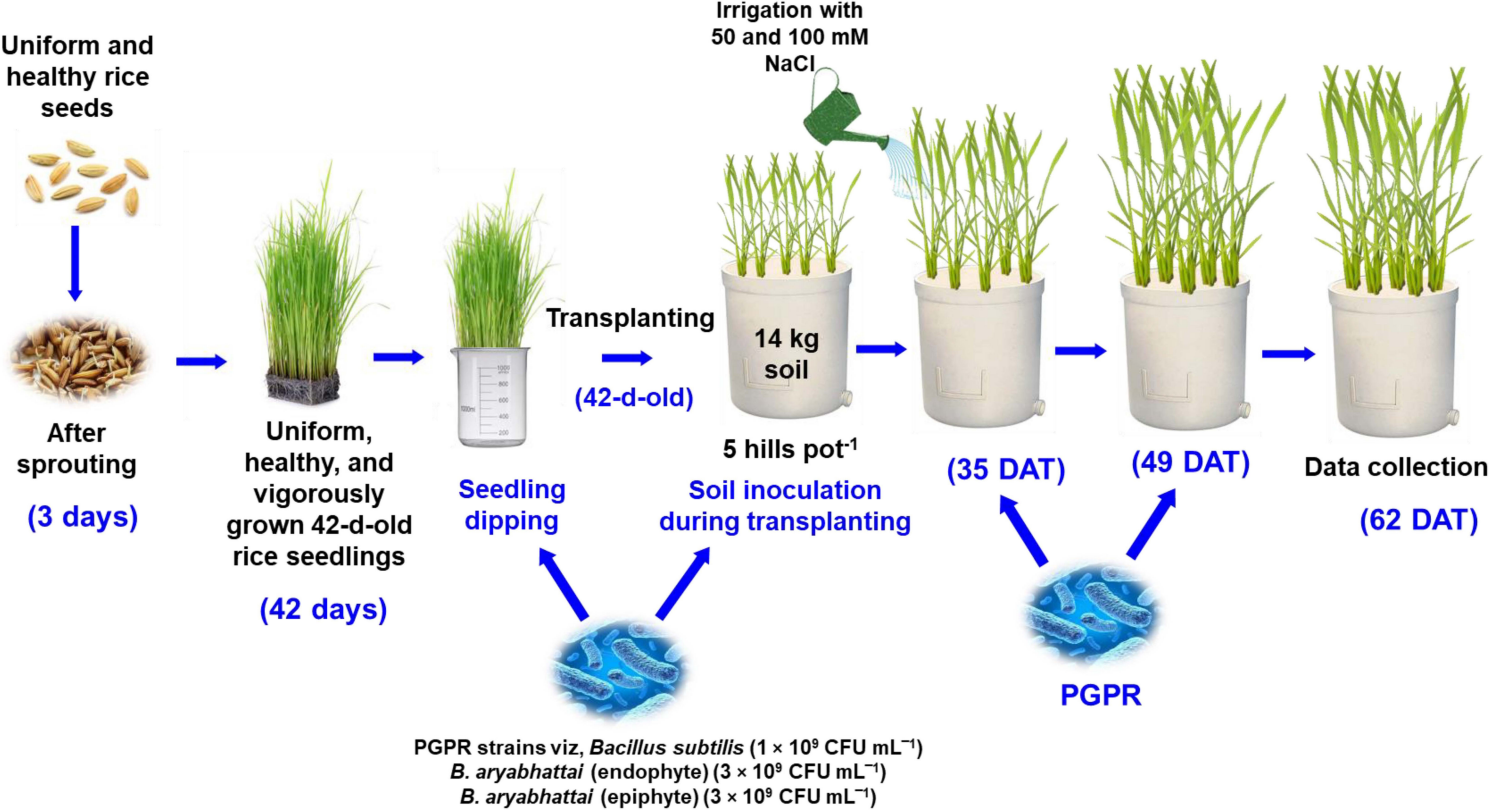
Schematic representation of rice plant growth conditions, salt stress impositions, and PGPR strain treatments.

Uniform and healthy seeds of a Zn-enriched rice variety (*Oryza sativa* cv. BRRI dhan100) containing a Zn content of 25.7 mg kg^−1^ were used in this experiment. Vigorously growing, uniform, and disease-free 42-day-old seedlings were then transplanted into Wagner pots (14 L) with soil containing BRRI (2020) recommended fertilizer doses (Urea: 138 kg ha^‾1^, TSP: 51 kg ha^‾1^, MoP: 63 kg ha^‾1^, Gypsum: 60 kg ha^‾1^, and ZnSO_4_: 4 kg ha^‾1^). Five hills in each pot were maintained at a uniform distance until the reproductive stage and then thinned to two hills per pot (**Figure 1**). Three different PGPR suspensions were applied using seedling dipping and soil drenching methods: endophytic *Bacillus subtilis* (1 × 10^9^ CFU mL^‾1^), endophytic *B*. *aryabhattai* (3 × 10^9^ CFU mL^‾1^) and epiphytic *B*. *aryabhattai* (3 × 10^9^ CFU mL^‾1^). The applications were made at three distinct growth stages: transplantation of 42-day-old seedlings, vegetative stage at five weeks post-transplantation, and panicle initiation stage at seven weeks post-transplantation. Five weeks after transplantation, the plants were irrigated twice with 50 mM and 100 mM NaCl solutions at seven-day intervals, whereas the control group was irrigated with only water. The experiment was conducted as a completely randomized design (CRD) with three replications.

### 2.2 Measurements of crop growth attributes

Crop growth attributes (plant height, leaf area, plant fresh, and dry weight) were measured at 62 days after transplanting. Plant height was calculated by measuring the length of five plants per pot from the base to the most extended leaf tip and then averaging the measurements. Leaf area was measured from five randomly selected leaves per pot using a length-width method (Francis et al., 1969). Fresh weight (FW) was determined by gently uprooting five hills per pot and weighing them. The uprooted plants were then oven-dried for 72 h at 80°C, and the dry weight (DW) of each plant was measured. The data were presented as the averages of the five measurements.

### 2.3 Measurements of physiological and biochemical attributes

#### 2.3.1 Relative water content and proline content

Leaf relative water content (RWC) was determined by measuring the FW of rice leaf blades. The leaves were then placed in water for 12 h for determination of the turgid weight (TW) and later oven-dried (48 h, 80°C) for measurement of leaf DW. The RWC was determined using the formula: RWC (%) = (FW–DW)/(TW–DW) × 100 (Barrs and Weatherley, 1962). The leaf proline (Pro) content was determined with a spectrophotometer (GENESYS 10S UV-Vis, Thermo Fisher Scientific Inc., Madison WI, USA) using 0.5 g of leaf tissue and the method described by Bates et al. (1973).

#### 2.3.2 Ion content

Leaf Na^+^ and K^+^ contents were quantified using a portable ion meter (Horiba, Tokyo, Japan). Sap from fresh leaf samples was introduced into the calibrated sensor of the ion meter after rinsing the sensor with deionized water to eliminate residual dirt.

#### 2.3.3 Chlorophyll content

For pigment extraction, 0.25 g of fresh leaf tissue from plants from each treatment was chopped and immersed in a water bath with 10 mL of 100% ethanol at 70°C until they turned white. The colored chlorophyll (Chl) chromophore was then measured spectrophotometrically at wavelengths of 663, 645, and 470 nm. The concentrations of Chl *a*, Chl *b*, and Chl (*a*+*b*) were determined using the method described by Arnon (1949).

#### 2.3.4 Stomatal conductance

Stomatal conductance (*g_s_*) was quantified from the surfaces of fully expanded leaves of individual plants from all experimental treatments using a leaf porometer (model SC-1, Decagon Devices, Inc., Pullman, WA, USA).

#### 2.3.5 Chlorophyll fluorescence

A fluorimeter (Pocket PEA Chlorophyll Fluorimeter, Hansatech Instruments Ltd., Norfolk, UK) was employed to measure the Chl fluorescence of fully expanded leaf blades. The minimum fluorescence (*F_o_*) was recorded in a simulated dark condition using clips. The maximum fluorescence (*F_m_*) was obtained 15 min later by giving a light pulse of 3000 μmol m^-2^ s^-1^. The photosystem II (PSII) activities were calculated using the following equation: *F_v_*/*F_m_* = (*F_m_*–*F_o_*)/*F_m_* where the variable fluroscence is denoted by F_*v*_.

#### 2.3.6 Indole-3-acetic acid concentration

The concentration of IAA was quantified using previously described methods (Gordon and Weber, 1951). Extracts were prepared from 0.5 g leaf material by grinding in an ice-cooled mortar and pestle in 2 mL 80% cold methanol, followed by centrifugation at 5,000×g for 5 min at 4°C. A 2 mL volume of Salkowski reagent (2% 0.5 M FeCl_3_ in 35% HClO_4_) was then mixed with 1 mL of the supernatant and 2 drops of orthophosphoric acid. Two hours later, the optical density of the solution was measured spectrophotometrically at 530 nm. The IAA concentrations in the samples were determined using an IAA standard curve.

### 2.4 Estimation of oxidative stress indicators: malondialdehyde, hydrogen peroxide content, and electrolyte leakage (%)

The leaf malondialdehyde (MDA) content was quantified following the method of Heath and Packer (1968), with a slight modification (Hasanuzzaman et al., 2022a). A reaction mixture was prepared by mixing 4 mL of thiobarbituric acid (TBA) reagent (20% TCA + 0.5% TBA) reagent with 1 mL of supernatant. The supernatant was prepared by homogenizing leaf tissues (0.5 g) with 3 mL of 5% trichloroacetic acid (TCA) and centrifuging it at 11,500×g for 10 min at 4°C. Then spectrophotometric absorbance was recorded at 532 and 600 nm after incubating the mixture in a water bath at 95 °C for 30 min and cooling it quickly on ice. The final MDA content was calculated using an extinction coefficient of 155 mM^-1^ cm^-1^. The method of Yang et al. (2007) was used to determine H_2_O_2_ content. The reaction mixture was prepared by adding 3 mL of 5% TCA to 0.5 g leaf material and centrifuging, followed by adding 1 ml of 1 M potassium iodide and 3 mL of 50 mM potassium phosphate (K-P) buffer (pH 7.0). The H_2_O_2_ content was calculated after spectrophotometric readings at 390 nm and using an extinction coefficient of 0.28 μM^-1^ cm^-1^. Electrolyte leakage (EL%) was measured following the method of Dionisio-Sese and Tobita (1998) and calculated using the following formula: EL = (EC_1_/EC_2_) × 100.

### 2.5 Quantification of ascorbate and glutathione content

Ascorbate (AsA) content was determined following the method of Nahar et al. (2016) by preparing leaf extracts in 1 mM ethylenediaminetetraacetic acid in 5% meta-phosphoric acid, centrifuging, mixing with 0.1 M dithiothreitol and distilled water, and neutralizing with 0.5 M K-P buffer (pH 7.0). The total and reduced AsA concentrations were measured spectrophotometrically at A_265_ and the dehydroascorbate (DHA) was calculated by subtracting the concentration of reduced AsA from the total AsA. The glutathione (GHS) content was determined by oxidizing the leaf extracts with 5,5-dithio-bis-2-nitrobenzoic acid and neutralizing with 0.5 M K-P buffer (pH 7.0) in the presence of reduced nicotinamide adenine dinucleotide phosphate (NADPH) and glutathione reductase (GR), followed by spectrophotometric measurement at A_412_. The oxidized glutathione (GSSG) content was measured by neutralizing the extract with 2-vinylpyridine and K-P buffer. The final GHS content was estimated by comparison to standard curves for GSH and GSSG (Hasanuzzaman et al., 2022a).

### 2.6 Enzyme extraction and protein measurement

Enzymes were extracted using a previously described method (Hasanuzzaman et al., 2022a), which involved grinding of 0.5 g leaf tissue in a precooled mortar pestle with an extraction buffer containing 50 mM K-P buffer (pH 7.0) in 1 mM AsA, 5mM β-mercaptoethanol, 10% glycerol, and 100 mM KCl solution. The resultant leaf homogenate was centrifuged for 12 min at 11,500×g at 4°C. The clear supernatant was used to determine antioxidant enzyme activities and the free protein content was determined using the method of Bradford (1976).

### 2.7 Antioxidant enzyme activity determinations

Ascorbate peroxidase (APX; EC: 1.11.1.11) activity was determined using the method of Nakano and Asada (1981) and an extinction coefficient of 2.8 mM^-1^ cm^-1^. Dehydroascorbate reductase (DHAR; EC: 1.8.5.1) activity was similarly assayed using an extinction coefficient of 14 mM^-1^ cm^-1^. The method of Hossain et al. (1984) and an extinction coefficient of 6.2 mM^-1^ cm^-1^ were used to determine the monodehydroascorbate reductase (MDHAR; EC: 1.6.5.4) activity. The method of Hasanuzzaman et al. (2022a) and an extinction coefficient of 6.2 mM^-1^ cm^-1^ were used to measure glutathione reductase (GR; EC: 1.6.4.2) activity.

The activities of glutathione peroxidase (GPX; EC: 1.11.1.9), glutathione-*S*-transferase (GST; EC: 2.5.1.18), and catalase (CAT; EC: 1.11.1.6) were also measured as described previously mentioned method (Hasanuzzaman et al., 2022a), with a slight modification from Elia et al. (2003) for GPX determination. The extinction coefficients for GPX, GST, and CAT were 6.62 mM^-1^ cm^-1^, 9.6 mM^-1^ cm^-1^, and 39.4 mM^-1^ cm^-1^, respectively. Lipoxygenase (LOX; EC: 1.13.11.12) activity was measured using the method by Doderer et al. (1992), with linolenic acid used as a substrate. The method of El-Shabrawi et al. (2010) was used to determine the superoxide dismutase (SOD; EC: 1.15.1.1) activity, using xanthine and xanthine oxidase as substrates. Peroxidase (POD; EC: 1.11.1.7) activity was determined following the method of Hemeda and Klein (1990).

### 2.8 Methylglyoxal content and glyoxalase enzyme activity determinations

The amount of methylglyoxal (MG) in leaf tissues was estimated using the method described by Wild et al. (2012). The leaf samples were homogenized with 5% perchloric acid, and the concentration of MG was determined by measuring the spectrophotometric absorbance at 288 mm and calculated using a standard curve. The activities of glyoxalase I (Gly I, EC: 4.4.1.5) and glyoxalase II (Gly II, EC: 3.1.2.6), were determined according to Hasanuzzaman et al. (2022a) and Principato et al. (1987) using extinction coefficients of 3.37 and 13.6 mM^-1^ cm^-1^, respectively.

### 2.9 Statistical analyses

The data were presented as the mean ± standard deviation of three replications. Tukey’s honestly significant difference (HSD) test at *p* ≤ 0.05 was used to separate means in the statistical analysis by applying the one-way analysis of variance (ANOVA) technique using the CoStat v.6.400 (2008) computer software.

## 3 Results

### 3.1 Effects on the growth attributes

**Figure 2:**
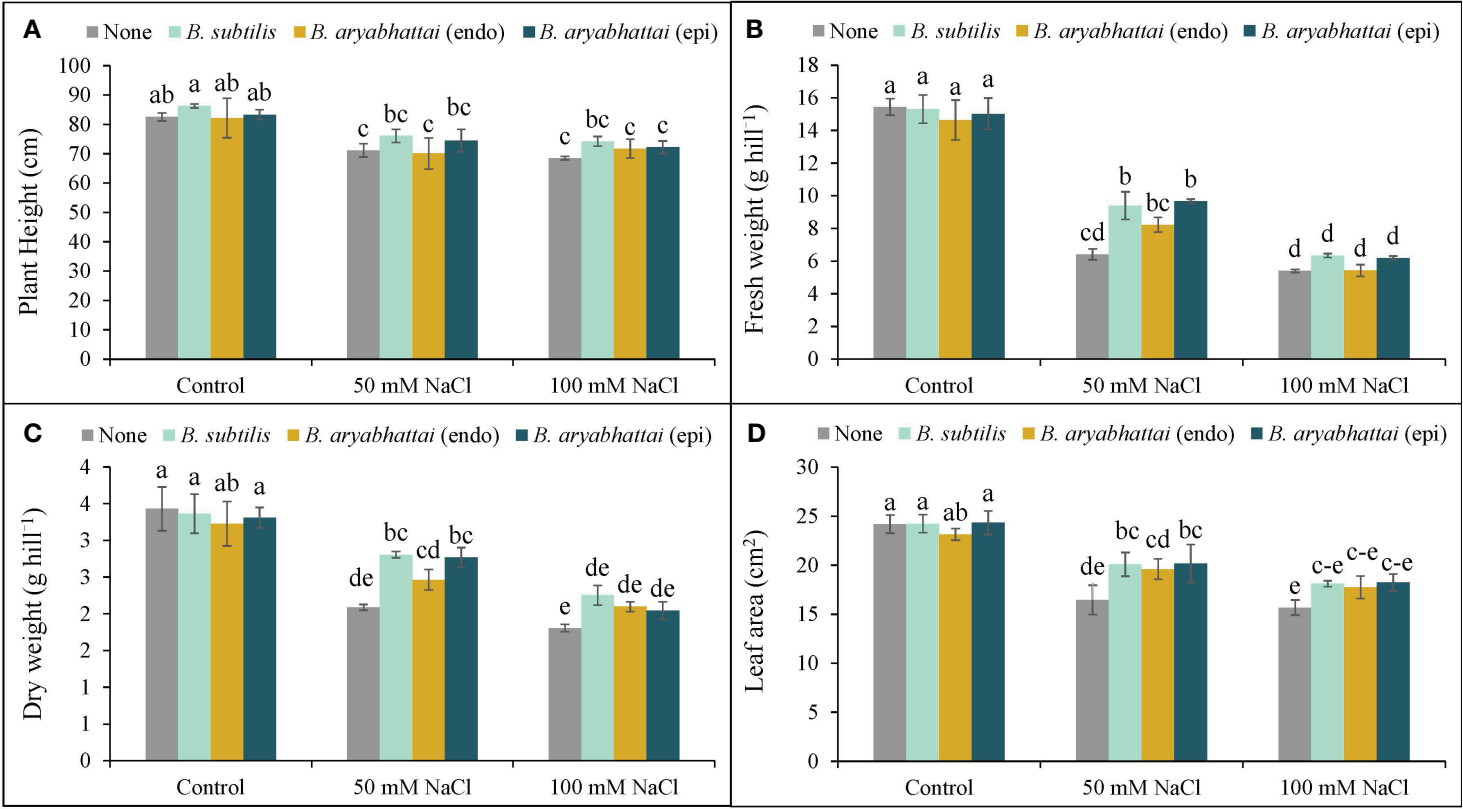
Variations in plant height **(A)**, fresh weight **(B)**, dry weight **(C)**, and leaf area **(D)** of rice plants under salt stress (50 or 100 mM NaCl) in the absence or presence of three PGPRs (*Bacillus subtilis*, epiphytic *B. aryabhattai*, and endophytic *B. aryabhattai*). Data are presented as mean ± standard deviation of three replications (*n*=3). Distinct letters on the bars show significant differences between treatments at *p* ≤ 0.05 from Tukey’s HSD test.

Plant height was reduced by 14 and 17% in response to 50 and 100 mM NaCl stress, respectively, when compared to the unstressed controls (no NaCl treatment). However, the application of *Bacillus subtilis* demonstrated superior performance than other strains by enhancing plant height significantly by 7 and 8% under 50 and 100 mM NaCl stress conditions, respectively, compared to the stressed alone plants. On the other hand, both the endophytic *B*. *aryabhattai* and epiphytic *B*. *aryabhattai* applications showed little to no change in plant height under similar stress conditions (**Figure 2A**).

In the presence of 50 and 100 mM NaCl stress, plant FW was decreased by 58 and 65%, respectively (**Figure 2B**), while the DW was declined by 39% and 47%, respectively, compared to the unstressed controls (**Figure 2C**). However, under 50 mM NaCl stress, treatment with *B. subtilis* (51%) and epiphytic *B. aryabhattai* (47%) led to a notable increase in FW compared to the non-inoculated plants, but this difference was not statistically significant under 100 mM NaCl stress (**Figure 2B**). Similarly, in terms of DW, both *B*. *subtilis* and epiphytic *B*. *aryabhattai* outperformed the endophytic *B*. *aryabhattai* in enhancing plant DW than the non-inoculated plants (**Figure 2C**).

Both salt stress levels significantly reduced the leaf area compared to unstressed controls (**Figure 2D**). Nonetheless, all PGPR strains were found to increase leaf area at both stress conditions but *B*. *subtilis* and epiphytic *B*. *aryabhattai* showed the greatest enhancements in leaf area by 22% and 19%, respectively, under only 50 mM salt stress (**Figure 2D**).

### 3.2 Effects on photosynthetic attributes

Chlorophyll *a* and Chl *b* contents in rice leaves were decreased significantly under both 50 and 100 mM NaCl stress conditions compared to the control (**Table 1**). This decline eventually led to the reduction of total Chl (*a*+*b*) content. However, salinity-stressed plants treated with PGPRs showed significantly increased amounts of photosynthetic pigment contents compared to non-treated plants under similar stress conditions. *B. subtilis* and epiphytic *B. aryabhattai* were most effective in restoring the Chl pigments in all cases specifically, under 100 mM NaCl stress. Moreover, among those PGPR strains, *B. subtilis* outperformed the latter by significantly enhancing Chl *a* (25%), Chl *b* (74%), and Chl (*a*+*b*) (43%) contents (**Table 1**). Though endophytic *B. aryabhattai* escalated the photosynthetic pigment contents under both stress levels than the non-inoculated plants, the increments were not as significant as the other PGPR strains (**Table 1**).

**Table 1:** Changes in photosynthetic attributes of rice plants under salt stress (S_1_ = 50 mM NaCl; S_2_ = 100 mM NaCl) in the absence or presence of three PGPRs (*Bacillus subtilis*, epiphytic *B. aryabhattai*, and endophytic *B. aryabhattai*).

Treatments	Chl *a* content (mg g^-1^ FW)	Chl *b* content (mg g^-1^ FW)	Chl (*a*+*b*) content (mg g^-1^ FW)	Stomatal conductance (mmol m^-2^s^-1^)	Chlorophyll fluorescence (F*_v_*/F*_m_*)
Control	1.34 ± 0.01 a	1.36 ± 0.10 a	2.70 ± 0.09 a	41.40 ± 1.50 a	0.77 ± 0.01 ab
*B. subtilis*	1.31 ± 0.01 a	1.49 ± 0.07 a	2.80 ± 0.09 a	40.90 ± 1.96 ab	0.76 ± 0.01 ab
*B. aryabhattai* (endo)	1.31 ± 0.00 a	1.35 ± 0.11 a	2.67 ± 0.11 ab	39.70 ± 0.58 abc	0.77 ± 0.01 ab
*B. aryabhattai* (epi)	1.33 ± 0.01 a	1.44 ± 0.07 a	2.78 ± 0.06 a	40.80 ± 1.50 ab	0.76 ± 0.01 ab
S_1_	1.11 ± 0.07 bc	0.79 ± 0.05 d	1.89 ± 0.11 ef	35.90 ± 0.92 de	0.73 ± 0.01 cd
S_1_+ *B. subtilis*	1.31 ± 0.02 a	1.11 ± 0.03 b	2.42 ± 0.05 bc	36.30 ± 0.23 cde	0.75 ± 0.02 bcd
S_1_+ *B. aryabhattai* (endo)	1.19 ± 0.09 ab	1.04 ± 0.06 bc	2.23 ± 0.08 cd	35.50 ± 0.72 de	0.76 ± 0.01 ab
S_1_+ *B. aryabhattai* (epi)	1.30 ± 0.03 a	1.07 ± 0.04 b	2.37 ± 0.07 c	35.85 ± 0.40 de	0.78 ± 0.00 ab
S_2_	0.90 ± 0.07 d	0.51 ± 0.04 e	1.41 ± 0.05 g	34.70 ± 0.69 e	0.72 ± 0.01 d
S_2_+ *B. subtilis*	1.13 ± 0.03 ab	0.88 ± 0.03 cd	2.01 ± 0.04 de	38.65 ± 0.40 a-d	0.76 ± 0.00 abc
S_2_+ *B. aryabhattai* (endo)	1.03 ± 0.09 cd	0.76 ± 0.04 d	1.79 ± 0.06 f	37.80 ± 0.81 a-d	0.77 ± 0.01 ab
S_2_+ *B. aryabhattai* (epi)	1.12 ± 0.02 bc	0.07 ± 0.05 d	1.94 ± 0.06 ef	36.90 ± 1.50 b-e	0.78 ± 0.01 a

Data are presented as mean ± standard deviation of three replications (n=3). Distinct letters on each column show significant differences between treatments at *p* ≤ 0.05 from Tukey’s HSD test.

Furthermore, *g_s_* was decreased in a dose-dependent manner with increased salinity levels compared to the unstressed controls. The addition of all three PGPRs resulted in only a negligible increment in *g_s_* under 50 mM NaCl stress compared to the salt-stressed plants (**Table 1**). Whereas, application of *B*. *subtilis* and epiphytic *B*. *aryabhattai*, increased the *g_s_* significantly by 10% and 9%, respectively, under 100 mM salt stress (**Table 1**). A notable reduction (7%) in the *F_v_*/*F_m_* ratio was observed when plants were subjected to 100 mM NaCl stress relative to the control (**Table 1**). Though all the PGPR treatments restored the ratio in both doses of salt stress, the increment by epiphytic *B. aryabhtattai* was significant (8%) in 100 mM NaCl stress than the non-inoculated plants.

### 3.3 Effect on the physiological attributes

#### 3.3.1 Osmotic adjustment and relative water content

**Figure 3:**
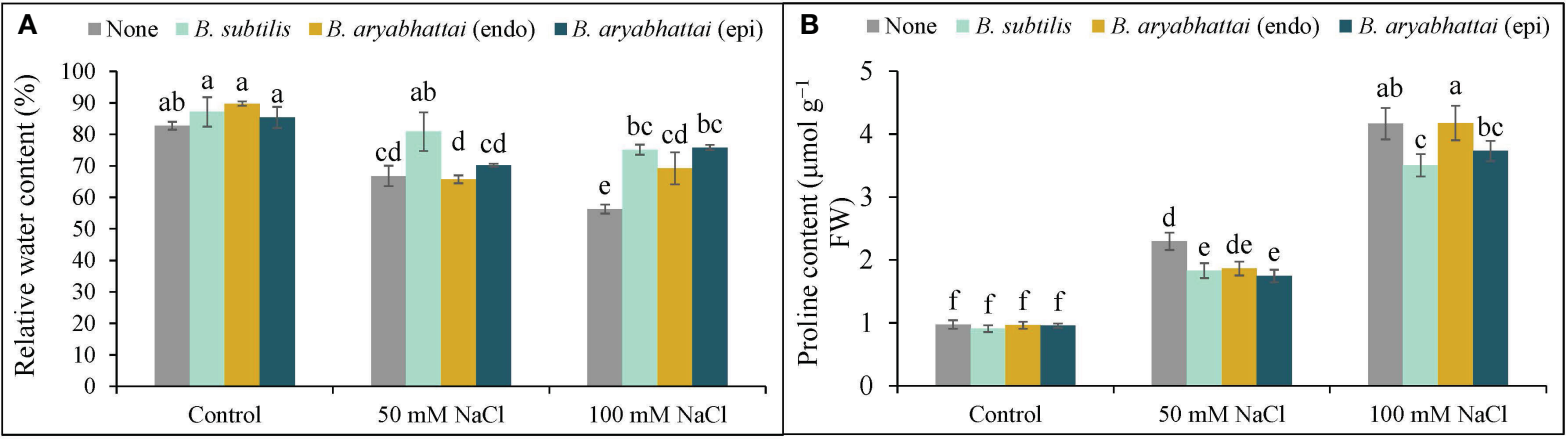
Changes in relative water content **(A)**, and proline content **(B)** of rice plants under salt stress (50 and 100 mM NaCl) in the absence or presence of three PGPRs (*Bacillus subtilis*, epiphytic *B*. *aryabhattai*, and endophytic *B*. *aryabhattai*). Data are presented as mean ± standard deviation of three replications (*n*=3). Distinct letters on the bars show significant differences between treatments at *p* ≤ 0.05 from Tukey’s HSD test.

The RWC was reduced under both 50 and 100 mM NaCl stress with a significant reduction (26%) under higher salinity dose compared to the unstressed controls (**Figure 3A**). However, PGPR treatments increased the RWC under both stress conditions, where, the improvement by *B*. *subtilis* was the highest (19%) under 100 mM NaCl stress compared to the salt-stressed controls (**Figure 3A**). Compared to the non-stressed controls, Pro content significantly increased in rice plants when exposed to increasing levels of salinity stress with the highest increment (327%) under 100 mM NaCl stress. The application of PGPR improved this condition by reducing the excessively generated Pro content in all treatments, where *B. subtilis* performed the best in reducing the Pro content (16%) compared to the salt-stressed controls under 100 mM salinity stress (**Figure 3B**).

#### 3.3.2 Ion homeostasis

**Figure 4:**
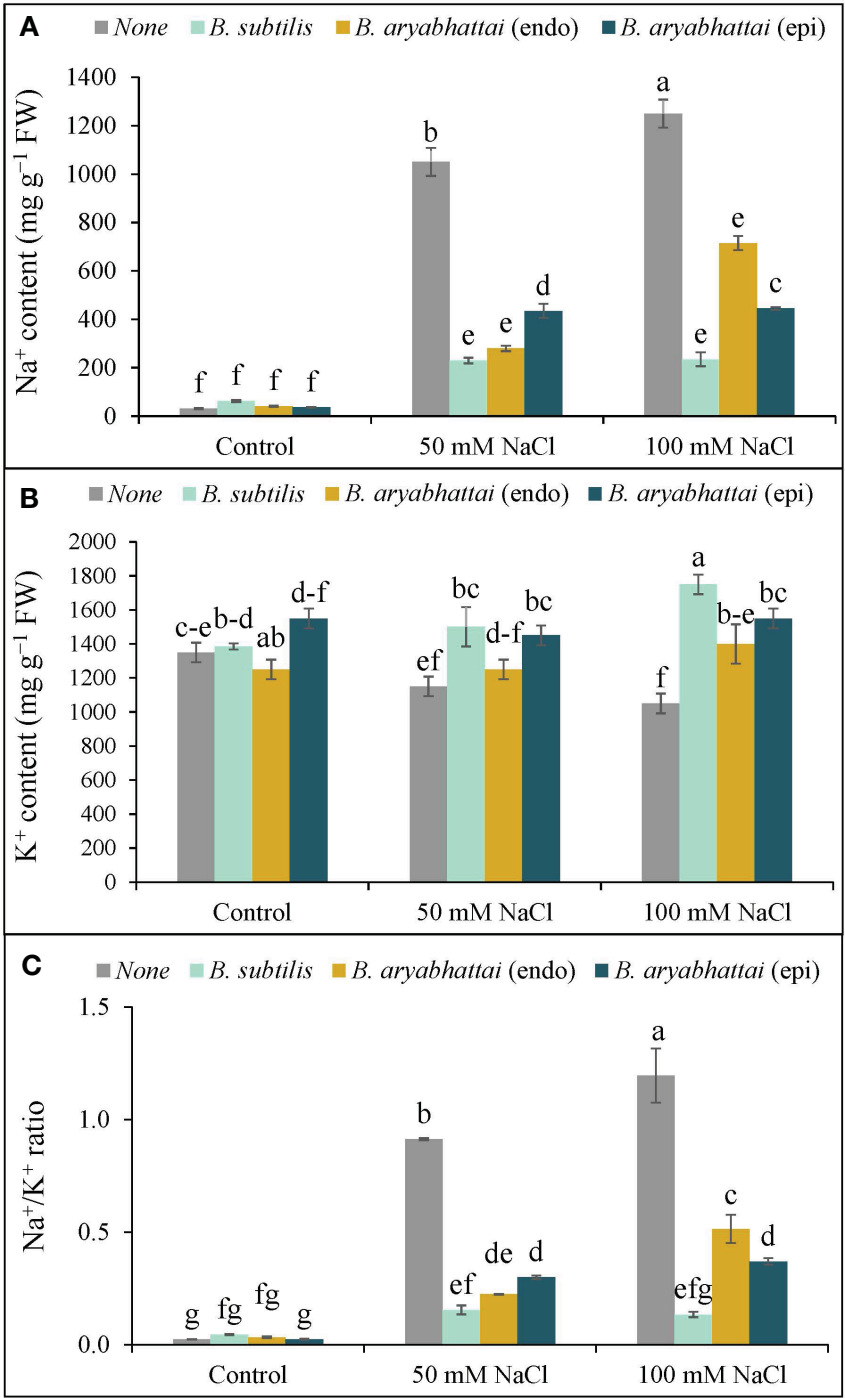
Variations in Na^+^ content **(A)**, K^+^ content **(B)**, and Na^+^/K^+^ ratio **(C)** of rice plants under salt stress (50 and 100 mM NaCl) in the absence or presence of three PGPRs (*Bacillus subtilis*, epiphytic *B*. *aryabhattai*, and endophytic *B*. *aryabhattai*). Data are presented as mean ± standard deviation of three replications (*n*=3). Distinct letters on the bars show significant differences between treatments at *p* ≤ 0.05 from Tukey’s HSD test.

The application of 50 and 100 mM NaCl stress disrupted the ion homeostasis in rice plants, as evidenced by increased Na^+^ accumulation as well as decreased K^+^ accumulation, resulting in a 40 and 53-fold increase in the Na^+^/K^+^ ratio, respectively, compared to control plants (**Figures 4A–C**). Nevertheless, PGPR treatments reversed this imbalance by preserving ion homeostasis by significantly reducing Na^+^ accumulation and enhancing K^+^ uptake through rice plant roots. Among them, the greatest reduction (81%) in Na^+^ was noted with *B. subtilis* inoculation under 100 mM NaCl stress, leading to a significant increase (67%) in K^+^ accumulation (**Figures 4A, B**), which restored the Na^+^/K^+^ ratio by nearly 89% (**Figure 4C**) compared to the stressed plants.

#### 3.3.3 Indole-3-acetic acid content

In comparison to the unstressed control, the concentration of IAA significantly decreased in rice plants exposed to increasing levels of salinity stress. Specifically, plants subjected to 100 mM NaCl stress demonstrated a significant IAA reduction (32%) compared to the controls (**Supplementary Figure 1**). However, the application of PGPRs ameliorated this condition by boosting the concentrations under both salinity conditions. Notably, among the three PGPRs, epiphytic *B. aryabhattai* was the most effective under both salinity levels, increasing IAA concentrations by approximately 49 and 92%, respectively, compared to stressed plants (**Supplementary Figure 1**).

### 3.4 Oxidative stress indicators

**Figure 5:**
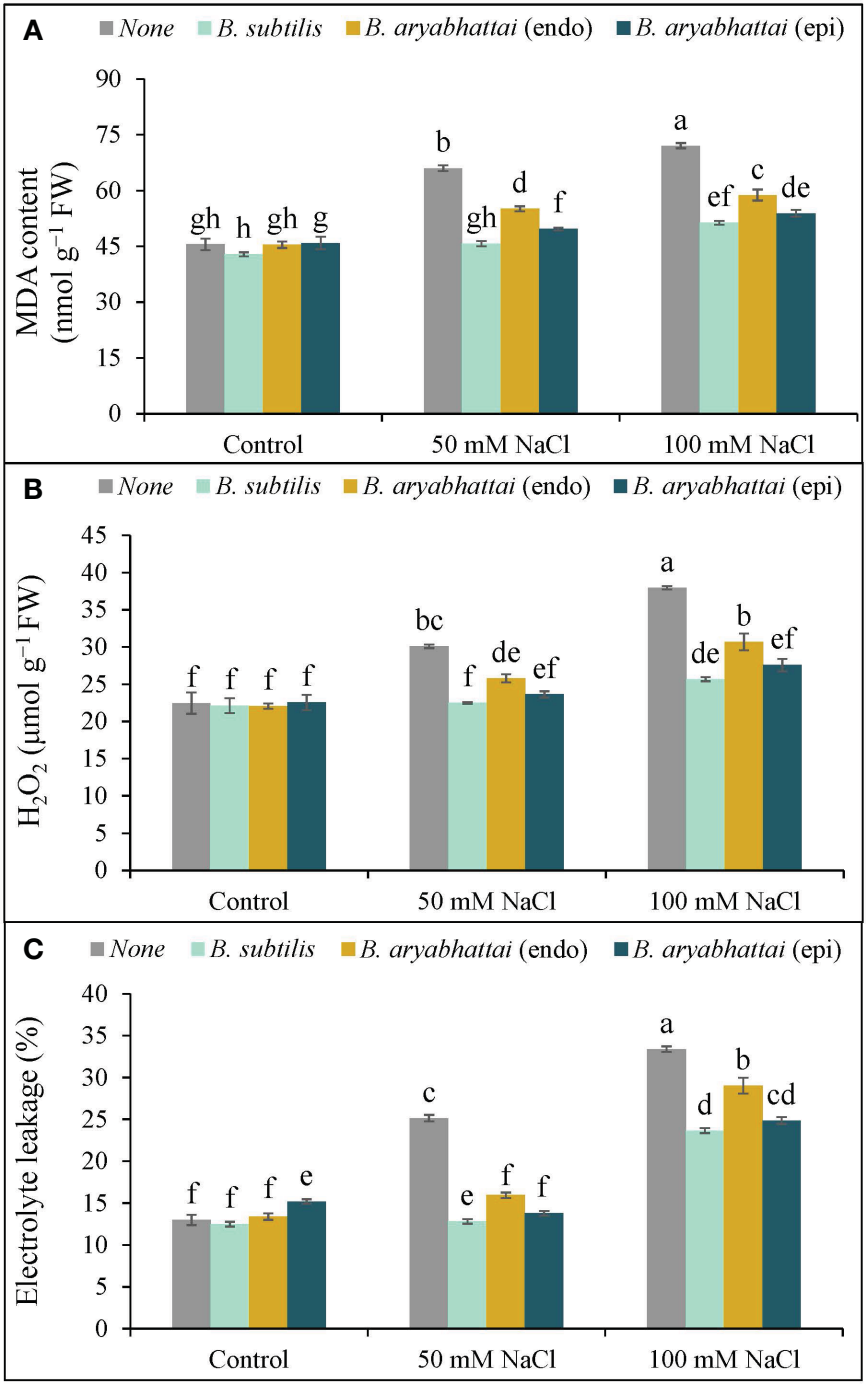
Variations in MDA content **(A)**, H_2_O_2_ content **(B)** and electrolyte leakage (%) **(C)** of rice plants under salt stress (50 and 100 mM NaCl) in the absence or presence of three PGPRs (*Bacillus subtilis*, epiphytic *B*. *aryabhattai*, and endophytic *B*. *aryabhattai*). Data are presented as mean ± standard deviation of three replications (*n*=3). Distinct letters on the bars show significant differences between treatments at *p* ≤ 0.05 from Tukey’s HSD test.

A significant rise in MDA content was observed with increasing salinity levels, where the highest (58%) lipid peroxidation was noted under 100 mM of NaCl stress compared to the controls (**Figure 5A**). Though PGPR treatment significantly reduced the MDA content in both stress conditions, *B. subtilis* outperformed other strains by reducing the MDA content by nearly 31 and 29% under 50 and 100 mM NaCl stress, respectively, compared to salt stress alone plants (**Figure 5A**).

Similarly, increasing levels of salinity doses corresponded with a rise in H_2_O_2_ levels, leading to membrane damage in rice plants. Under 100 mM NaCl stress, H_2_O_2_ levels rose substantially (69%) compared to the unstressed controls. However, PGPR treatment notably mitigated this effect with the greatest reduction (32%) in H_2_O_2_ level by *B. subtilis* under 100 mM NaCl stress, compared to the untreated plants (**Figure 5B**).

Likewise, EL% was also increased under increasing salinity levels, mirroring the trend observed in MDA and H_2_O_2_ contents. The highest EL% (20%) was noticed under 100 mM NaCl stress, which was almost double the leakage occurring in plants exposed to 50 mM NaCl salt stress compared to the unstressed controls (**Figure 5C**). Application of PGPR decreased the leakage significantly under 50 and 100 mM NaCl stress compared to the salt-stressed controls, where the highest decrease (12%) was noted with *B*. *subtilis* treatment under 50 mM NaCl salt stress (**Figure 5C**).

### 3.5 Effects on antioxidant defense systems

#### 3.5.1 AsA-GSH pools

**Figure 6:**
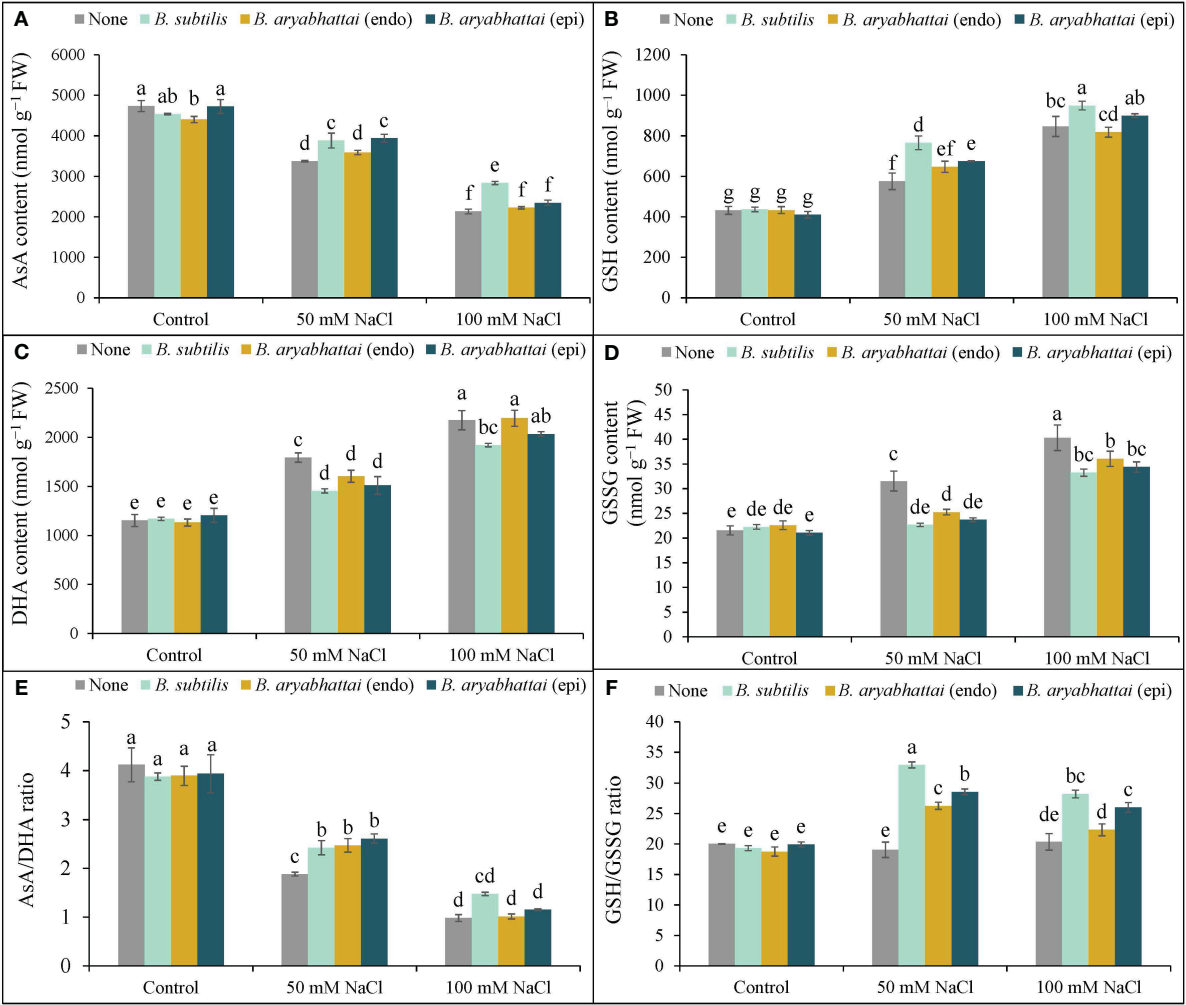
Variations in AsA content **(A)**, DHA content **(B)**, AsA/DHA ratio **(C)**, GSH content **(D)**, GSSG content **(E)** and GSH/GSSG ratio **(F)** of rice plants under salt stress (50 and 100 mM NaCl) in the absence or presence of three PGPRs (*Bacillus subtilis*, epiphytic *B*. *aryabhattai*, and endophytic *B*. *aryabhattai*). Data are presented as mean ± standard deviation of three replications (*n*=3). Distinct letters on the bars show significant differences between treatments at *p* ≤ 0.05 from Tukey’s HSD test.

Increasing salinity levels negatively affected AsA content with a significant reduction (53%) observed under 100 mM NaCl stress than the unstressed controls. However, the application of PGPRs mitigated this stress by increasing AsA content. *B. subtilis* was particularly effective than other PGPR strains, increasing AsA levels by 15 and 27% under 50 and 100 mM NaCl stress, respectively, than the non-inoculated controls (**Figure 6A**). The highest DHA content (89%) was observed under 100 mM NaCl stress and was approximately 1.5 times higher than that observed under 50 mM NaCl stress compared to the salt-stressed controls (**Figure 6B**). However, PGPRs ameliorated this effect, where *B. subtilis* notably reduced the DHA content (16%) at 100 mM NaCl stress than other strains compared to the salt-stressed controls (**Figure 6B**). Consequently, due to salt stress-induced reduction in AsA content and increase in DHA contents, the AsA/DHA ratio decreased than the non-stresses controls (**Figure 6C**). However, applying endophytic PGPRs restored the ratio under 50 and 100 mM NaCl stress, compared to plants only subjected to salt stress. Furthermore, among them, epiphytic *B. aryabhattai* increased the ratio (39%) under 50 mM NaCl stress, compared to the stressed controls. Except for *B. subtilis*, other PGPRs could not revert the increased AsA/DHA ratio under higher salinity levels (**Figure 6C**).

Compared to the control, GSH content increased by 33 and 94% under 50 and 100 mM NaCl stress, respectively (**Figure 6D**). The application of PGPRs further enhanced the GSH content under both salt stress conditions. The most substantial increase was found with *B. subtilis* application: a 25 and 12% increase at 50 and 100 mM saline conditions, compared to the salt-stressed plants only. The effect of other PGPRs was not significant at higher saline doses (**Figure 6D**). The level of GSSG content significantly increased by (87%) under 100 mM NaCl stress, compared to the controls (**Figure 6E**). However, PGPR treatments reduced the GSSG levels in salt-stressed plants. Application of *B*. *subtilis* gave the most significant reduction (28%) in 50 mM NaCl-stressed plants, compared to the salt-stressed controls (**Figure 6F**). However, the effect of PGPRs in reducing GSSG content was not significant at higher salt stress levels. The severity of the stress substantially decreased the GSH/GSSG ratio compared to the control. However, PGPR treatment recovered the GSH/GSSG ratio in salt-stressed rice plants with the most significant improvement (73%) in the ratio observed at 50 mM NaCl stress with the *B. subtilis* application (**Figure 6F**). The epiphytic PGPR, *B. aryabhattai*, performed better in increasing the GSH/GSSG ratio under both salt-stressed conditions, improving by nearly 50 and 28% under 50 and 100 mM NaCl stress, respectively. However, the endophytic PGPR, *B. aryabhattai*, was not as effective in reverting the GSH/GSSG ratio at both salinity stress levels, increasing the ratio by nearly 38% at 50 mM NaCl stress but showing a 3-fold lesser reduction under higher salinity stress. Therefore, among the three PGPRs, *B. subtilis* was most effective in restoring the AsA-GSH pool of salt-induced rice plants.

#### 3.5.2 Antioxidant enzyme activities

**Figure 7:**
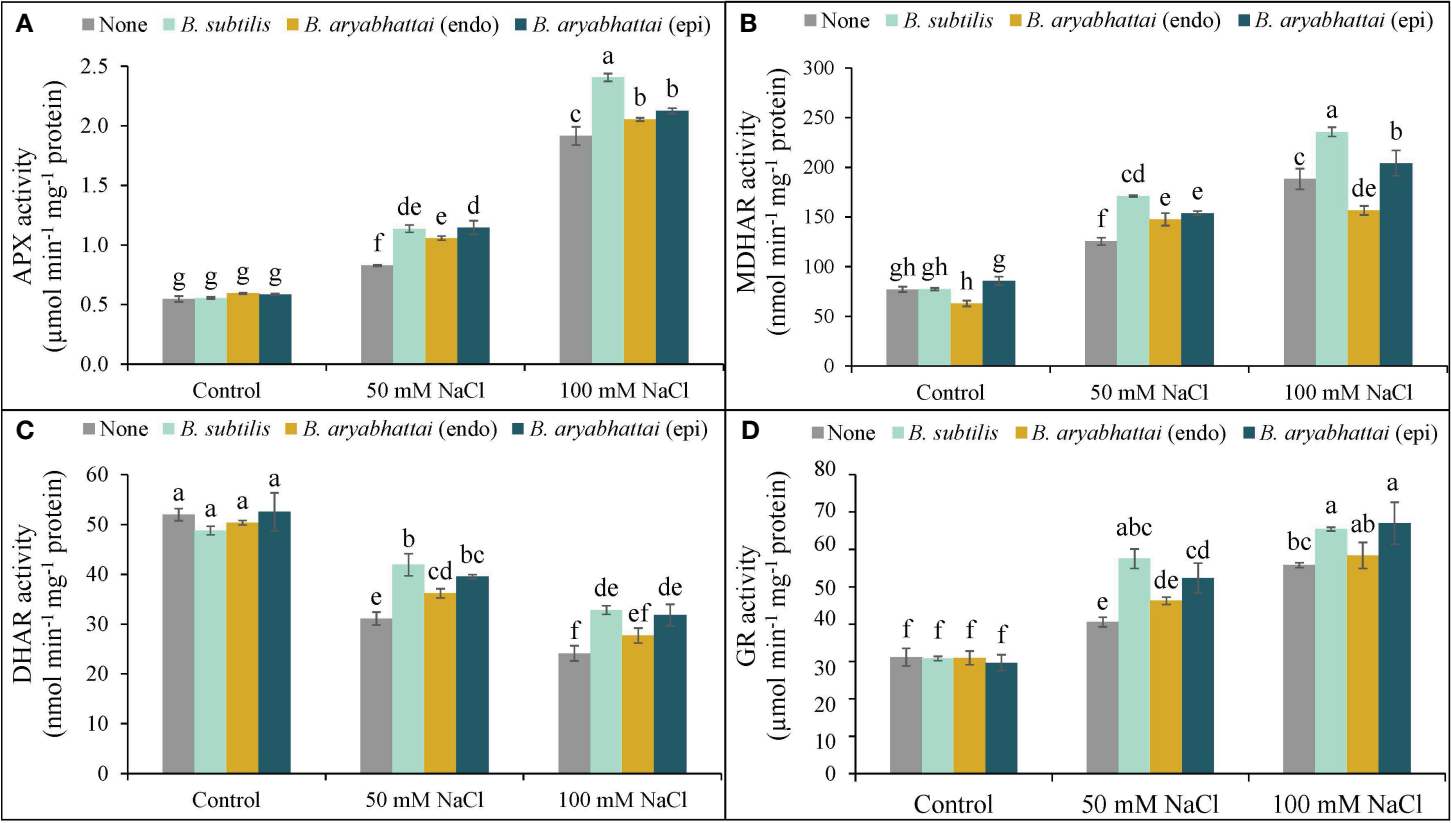
Changes in the activities of APX **(A)**, MDHAR **(B)**, DHAR **(C)**, and GR **(D)** of rice plants under salt stress (50 and 100 mM NaCl) in the absence or presence of three PGPRs (*Bacillus subtilis*, epiphytic *B*. *aryabhattai*, and endophytic *B*. *aryabhattai*). Data are presented as mean ± standard deviation of three replications (*n*=3). Distinct letters on the bars show significant differences between treatments at *p* ≤ 0.05 from Tukey’s HSD test.

A rise in APX activity was observed following the exposure to two different salinity levels with the most significant increase (250%) found under 100 mM NaCl stress, compared to controls, and was further increased by the application of PGPRs (**Figure 7A**). However, *B. subtilis* showed the best result among the other PGPRs, under higher salinity level by improving the APX activity by 26% than the stressed plants alone (**Figure 7A**). Similarly, MDHAR activity was also increased by 63 and 144% to the control under two different salinity doses, and further improvements were also noticed when rice plants were treated with three different PGPRs. However, among them, likewise APX activity, *B. subtilis* further improved the MDHAR activity (25%) than the salt-stressed alone plants (**Figure 7B**). A similar trend was also noticed in terms of the rise in GR activity which was then further enhanced by the application of *B. subtilis.* However, here, both *B. subtilis* and epiphytic *B. aryabhattai* performed a significant role in increasing GR activity by 17 and 20%, respectively under 100 mM NaCl stress (**Figure 7D**). On the other hand, DHAR activity was noticeably reduced under 50 and 100 mM NaCl stress, compared to the unstressed controls (**Figure 7C**). But, in this case, both *B. subtilis* and epiphytic *B. aryabhattai* showed a significant acceleration in DHAR activity than the stressed plants with the highest increment (35%) by *B. subtilis* at 50 mM NaCl compared to the salt-stressed controls (**Figure 7C**).

**Figure 8:**
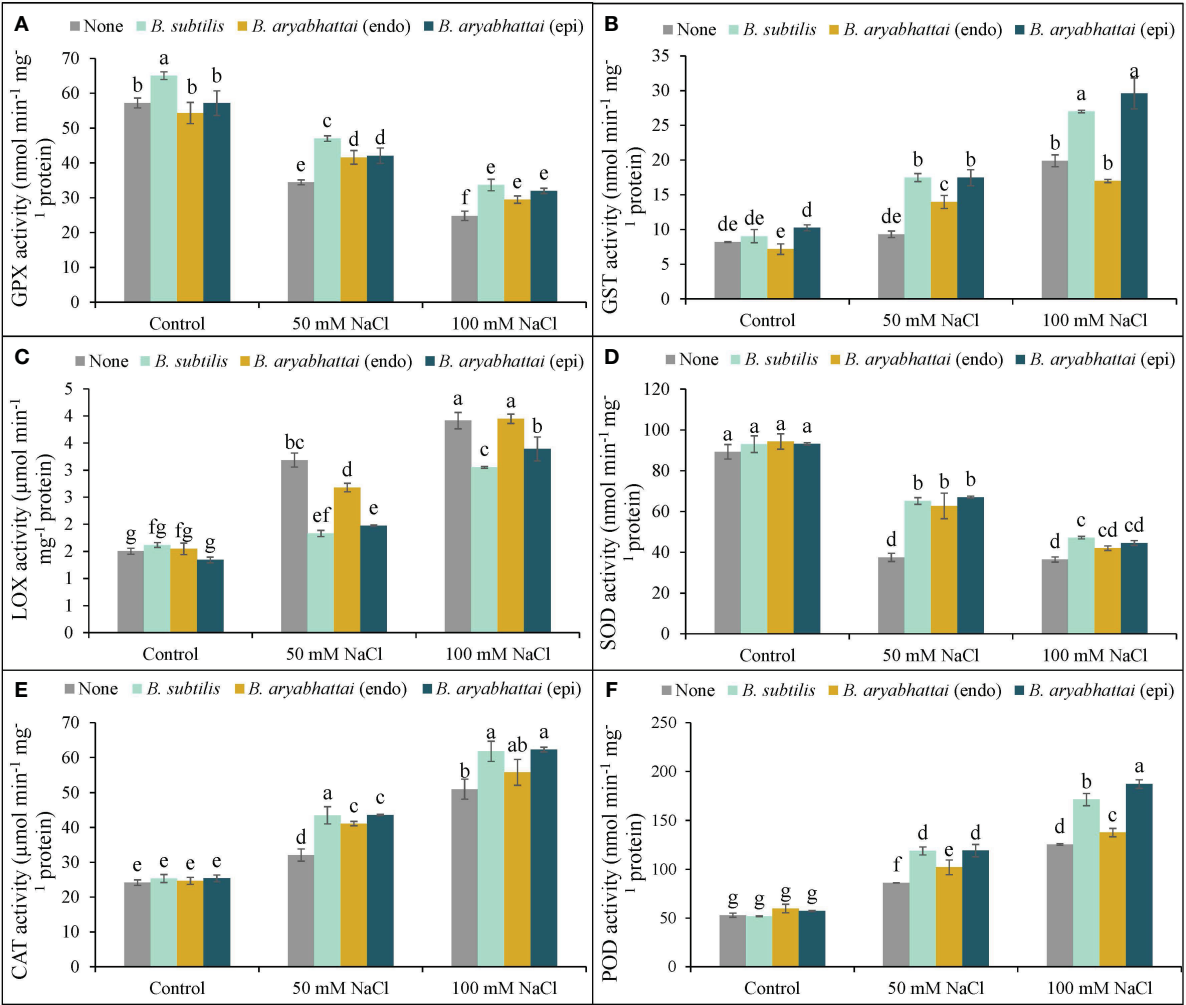
Variations in GPX **(A)**, GST **(B)**, LOX **(C)**, SOD **(D)**, CAT **(E)**, and POD **(F)** activities of rice plants under salt stress (50 and 100 mM NaCl) in the absence or presence of three PGPRs (*Bacillus subtilis*, epiphytic *B*. *aryabhattai*, and endophytic *B*. *aryabhattai*). Data are presented as mean ± standard deviation of three replications (*n*=3). Distinct letters on the bars show significant differences between treatments at *p* ≤ 0.05 from Tukey’s HSD test.

Rice plants exposed to two different salinity levels showed a notable reduction in the activities of GPX and SOD relative to the controls (**Figures 8A, D**). The application of PGPRs reverted this situation by increasing both antioxidant enzyme activities but the performance was better under the lower salinity dose. However, as previously found, a similar trend of the better activity of *B. subtilis* was also noticed for GPX, where the improvement was 37% than the stressed alone plants under 50 mM NaCl (**Figure 8A**). In terms of SOD, all three PGPRs performances were significantly similar under both stress conditions (**Figure 8D**).

On the other hand, the application of PGPRs on salt-stressed rice plants had notable positive effects in terms of the other antioxidant enzymes, e.g., GST, LOX, CAT, and POD. The highest increment of GST, LOX, CAT, and POD activities was found by nearly 143, 160, 111, and 137%, respectively, under 100 mM NaCl stress in rice plants than the non-stressed controls (**Figures 8B–F**). Nevertheless, the PGPRs application further boosted their activities (GST, CAT, and POD), and here, both *B. subtilis* and epiphytic *B. aryabhattai* were found to have almost similar significant positive results. Additionally, both of these PGPR strains performed better in reducing the LOX activity by 43 and 38% under 50 mM NaCl stress, where the levels were prominently increased by 112 and 160% with increasing salinity doses (**Figure 8C**).

### 3.6 Glyoxalase system

Salt stress also affected the glyoxalase system of rice plants which was evident with the highest (59%) increase in MG content under 100 mM NaCl stress compared to the controls (**Supplementary Figure 2A**). However, *B. subtilis* along with the endophytic and epiphytic *B. aryabhattai* changed this situation by reducing the MG content, though, in this case, epiphytic *B. aryabhattai* performed better under 100 mM NaCl stress by reducing it by 39% than the stressed alone rice plants (**Supplementary Figure 2A**). On the other hand, the activities of Gly I and Gly II were sharply reduced under salt stress (**Supplementary Figures 2B, C**) in contrast with the control plants. However, both *B. subtilis* and epiphytic *B. aryabhattai* showed statistically similar results in boosting the Gly I and Gly II activities under two different salt stress levels (**Supplementary Figures 2B, C**).

## 4 Discussion

The initial response of plants to salinity stress involves osmotic shock and ionic imbalances, which disrupt water uptake, break down cell membranes, and inhibit stomatal opening; ultimately restrict cell division, cell enlargement, photosynthesis, plant growth, and development (Rajabi et al., 2024). In this experiment, salinity-induced decreases in photosynthetic attributes (**Table 1**) and increases in lipid peroxidation (**Figure 5**) resulted in a reduction in plant growth parameters (**Figure 2**). However, application of PGPR strains alleviated salt stress and improved plant growth parameters by restoring photosynthetic efficiency and safeguarding the cell membranes. These improvements can be linked to PGPR-induced synthesis of IAA (**Supplementary Figure 1**). Auxin/IAA induces a variety of morphophysiological changes, such as increased root length, root surface area, nutrient uptake, and photosynthesis (Li et al., 2020; Iqbal et al., 2023). Moreover, microbial solubilization of iron (Rahimi et al., 2020) and magnesium (Ullah et al., 2022), combined with the stress-induced synthesis of siderophores by PGPRs may have led to the regeneration of the photosynthetic pigments, as well as restoration of F*_v_*/F*_m_* and g*_s_*. Our findings concur with those of Wang et al. (2023), who reported that PGPR application improved the photosynthetic efficiency of rice under salt stress. Although siderophore synthesis by the PGPR strains was not investigated in this current experiment, previous studies (Sultana et al., 2021; Ghazy and El-Nahrawy, 2021) have provided evidence that these three *Bacillus* strains are capable of synthesizing siderophores. All these responses contribute to improving plant growth in stressful environments, in agreement with the findings of Shultana et al. (2020) and Shultana et al. (2021).

Salinity-induced osmotic and ionic stresses create imbalances in the ion homeostasis of plant cells (Hu et al., 2022; Zhao et al., 2023), as confirmed in the present study by the elevated Na^+^/K^+^ ratio. However, PGPR application restored the ionic and osmotic balance by decreasing Na^+^ accumulation and increasing K^+^ absorption by the roots (**Figure 4**), and by reducing Pro accumulation and enhancing RWC (**Figure 3**). One explanation could be that bacterial EPS obstructs Na^+^ deposition on plant root surfaces (Shultana et al., 2020). These findings align with the study of Ji et al. (2022), which highlighted how wheat seedlings inoculated with PGPR under salt stress could stave off osmotic stress by regulating Pro and soluble sugar accumulation.

Excessively produced ROS induces oxidative stress in plants which is the secondary effect of salt stress. In this experiment, rice plants showed clear symptoms of salt stress-induced oxidative stress by increasing the stress indicators (**Figure 5**). However, to counteract the potential for ROS-induced damage, plants possess an intrinsic antioxidant defense mechanism containing enzymatic and non-enzymatic antioxidants, which is highly effective in preventing ROS production and regulating homeostasis, thereby safeguarding plant cells from oxidative damage (Rajabi et al., 2024; Yang et al., 2024). In this experiment, the balance between non-enzymatic antioxidants (AsA/DHA and GSH/GSSG) ratios in the AsA-GSH pool was disrupted (**Figure 6**) due to salt-induced oxidative stress which matches the results of other studies (Soliman et al., 2020; Zhu et al., 2020). However, the application of PGPR restored the ratios, suggesting ROS detoxification under salt stress, in agreement with the results of Puthiyottil and Akkara (2021).

In addition to non-enzymatic antioxidants, plants also possess antioxidant enzymes, such as APX, DHAR, MDHAR, and GR, which catalyze crucial reactions to detoxify ROS and maintain the AsA-GSH pool under stress (Kanwal et al., 2024). In the present study, salt stress disrupted these enzyme activities, but PGPR application ameliorated the salt-induced oxidative stress by stimulating them (**Figure 7**). Similar results have also been reported by Ali et al. (2022) in rice under salt stress. Moreover, in the present study, increased CAT and POD activities and reduced SOD activities were noted under salt stress, possibly indicating preferential ROS scavenging and regulation of −OH^•^ radical formation, also reported previously (Hu, 2019; Mubeen et al., 2022). However, PGPR application enhanced SOD activity, as well as CAT and POD (**Figure 8**), which further supports the findings of Hu (2019) in wheat under salt stress. Glutathione peroxidase uses GSH and thioredoxins to detoxify H_2_O_2_ as part of the non-heme group of POD, indicating the benefits of upregulating GPX activities under stress (Hasanuzzaman et al., 2022b). The present experiment showed a clear increment in GPX activity after the application of PGPR strains in salt-treated rice plants, with *B*. *subtilis* showing the most significant effect, in agreement with the study by Hasanuzzaman et al. (2022b). On the contrary, epiphytic *B*. *aryabhattai* was the most effective PGPR at increasing GST activity, in agreement with findings by Shultana et al. (2021), indicating its potential as a modulator of antioxidant enzyme activities in rice under salt stress.

Plants produce a certain amount of MG under normal conditions as well, but the production increases under stress. Methylglyoxal detoxification by the glyoxalase systems occurs with the help of the GSH enzyme, which converts MG into S-D-lactoylglutathione (SLG) using Gly I, followed by the breakdown of SLG into D-lactic acid by Gly II (Hasanuzzaman et al., 2022b). The current study showed a trend toward elevated MG production under salt stress, coupled with reductions in Gly I and Gly II activities (**Supplementary Figure 2**), in agreement with the findings of a previous study (Alabdallah et al., 2024). However, application of PGPR strains increased the level of GSH, thereby detoxifying MG by enhancing Gly I and Gly II activities, as previously reported by Kapadia et al. (2022).

Taken together, the findings presented here for salt-stressed rice plants clearly indicate that PGPR strains have the potential to ameliorate salt stress in rice by enhancing antioxidant enzyme activities and regulating key cellular biochemical pathways. However, the efficacy of PGPR may depend on many other factors, such as the plant species or variety, stress types and intensity, and bacterial strain characteristics. The specific mechanism underlying the salt tolerance conferred by PGPR is also unclear and remains largely unanswered. The findings presented here for PGPR effects on salt tolerance in rice plants highlight the usefulness of PGPR in sustainable agriculture and the need for more research on the complex mechanisms underlying the capacity of PGPR to mitigate salinity.

## 5 Conclusions

Our study comprehensively evaluated the impact of salinity stress on various morphophysiological attributes of rice plants and highlighted significant reductions in growth, photosynthetic efficiency, and hormonal regulation, along with increased oxidative damage and ionic imbalance, as key features of salt stress in rice. The application of PGPR showed encouraging and promising potential for alleviating the detrimental effects of salt stress on rice. Specifically, PGPR treatment enhanced nutrient uptake, bolstered hormone synthesis, restored ionic equilibrium, and bolstered antioxidant defenses, culminating in notable improvements in plant growth. Notably, among the tested PGPR strains, *Bacillus subtilis* emerged as particularly effective in mitigating salinity-induced toxicity and boosting plant tolerance. *B. aryabhattai*, as both an endophyte and an epiphyte, demonstrated commendable effects in enhancing rice plant resilience to salt stress; however, *B*. *subtilis* set a benchmark for efficacy. These findings underscore the practical applicability of PGPR in sustainable agriculture and the need for further investigation into the intricate mechanisms underpinning their salinity-mitigating properties and their potential impacts on grain quality enhancement under saline conditions. Moving forward, field trials focusing on incorporating PGPR inoculation, particularly in conjunction with salt-tolerant rice varieties are needed, for elucidating their precise effects on yield-contributing parameters and economic benefits. Such studies will thereby, advance our understanding and application of these beneficial microbial agents in saline environments.

## Data availability statement

The original contributions presented in the study are included in the article/**Supplementary Material**. Further inquiries can be directed to the corresponding authors.

## Author contributions

AS: Data curation, Investigation, Writing – original draft. AR: Investigation, Methodology, Writing – review & editing. SN: Methodology, Resources, Writing – review & editing. AK: Writing – review & editing. MK: Methodology, Resources, Supervision, Writing – review & editing. PP: Conceptualization, Funding acquisition, Resources, Writing – review & editing. MH: Conceptualization, Formal analysis, Funding acquisition, Investigation, Methodology, Project administration, Supervision, Visualization, Writing – original draft, Writing – review & editing.

## References

[B1] Alabdallah N. M. Al-Shammari A. S. Saleem K. AlZahrani S. S. Raza A. Asghar M. A.. (2024). Unveiling the mechanisms of silicon-induced salinity stress tolerance in *Panicum turgidum*: insights from antioxidant defense system and comprehensive metabolic and nutritional profiling. S. Afr. J. Bot. 168, 328–339. doi: 10.1016/j.sajb.2024.03.006

[B2] Ali Q. Ayaz M. Mu G. Hussain A. Yuanyuan Q. Yu C.. (2022). Revealing plant growth-promoting mechanisms of *Bacillus* strains in elevating rice growth and its interaction with salt stress. Front. Plant Sci. 13, 994902. doi: 10.3389/fpls.2022.994902 36119605 PMC9479341

[B3] Arnon D. T. (1949). Copper enzymes in isolated chloroplasts. Polyphenoloxidase. *Beta vulgaris* . J. Plant Physiol. 24, 1–15. 10.1104/pp.24.1.1 PMC437905 16654194

[B4] Barrs H. D. Weatherley P. E. (1962). A re-examination of the relative turgidity technique for estimating water deficits in leaves. Aust. J. Biol. Sci. 15, 413–428. doi: 10.1071/BI9620413

[B5] Basit F. Abbas S. Zhu M. Tanwir K. El-Keblawy A. Sheteiwy M. S.. (2023). Ascorbic acid and selenium nanoparticles synergistically interplay in chromium stress mitigation in rice seedlings by regulating oxidative stress indicators and antioxidant defense mechanism. Environ. Sci. pollut. Res. 30, 120044–120062. doi: 10.1007/s11356-023-30625-2 37936030

[B6] Bates L. S. Waldren R. P. Teari D. (1973). Rapid determination of free proline for water stress studies. Plant Soil 39, 205–207. doi: 10.1007/BF00018060

[B7] Bradford M. M. (1976). A rapid and sensitive method for the quantitation of microgram quantities of protein utilizing the principle of protein-dye binding. Anal. Biochem. 72, 248–254. doi: 10.1016/0003-2697(76)90527-3 942051

[B8] BRRI (Bangladesh Rice Research Institute) (2020). Adhunik dhaner chash. Joydebpur, (Gazipur: Bangladesh Rice Research Institute) Vol. 1701. 106.

[B9] Chakraborty M. Mahmud N. U. Ullah C. Rahman M. Islam T. (2021). Biological and biorational management of blast diseases in cereals caused by *Magnaporthe oryzae* . Crit. Rev. Biotechnol. 41, 994–1022. doi: 10.1080/07388551.2021.1898325 34006149

[B10] CoStat (2008). CoStat-Statistics Software, Version 6.400; CoHort Software (Monterey, CA, USA).

[B11] Dame Z. T. Rahman M. Islam T. (2021). *Bacilli* as sources of agrobiotechnology: recent advances and future directions. Green Chem. Lett. Rev. 14, 246–271. doi: 10.1080/17518253.2021.1905080

[B12] Dionisio-Sese M. L. Tobita S. (1998). Antioxidant responses of rice seedlings to salinity stress. Plant Sci. 135, 1–9. doi: 10.1016/S0168-9452(98)00025-9

[B13] Doderer A. Kokkelink I. van der Veen S. Valk B. Schram A. Douma A. (1992). Purification and characterization of two lipoxygenase isoenzymes from germinating barley. Biochim. Biophys. Acta Protein Struct. Mol. Enzymol. 112, 97–104. doi: 10.1016/0167-4838(92)90429-H 1554746

[B14] Elia A. C. Galarini R. Taticchi M. I. Dorr A. J. M. Mantilacci L. (2003). Antioxidant responses and bioaccumulation in *Ictalurus melas* under mercury exposure. Ecotoxicol. Environ. Saf. 55, 162–167. doi: 10.1016/S0147-6513(02)00123-9 12742363

[B15] El-Shabrawi H. Kumar B. Kaul T. Reddy M. K. Singla-Pareek S. L. Sopory S. K. (2010). Redox homeostasis, antioxidant defense, and methylglyoxal detoxification as markers for salt tolerance in Pokkali rice. Protoplasma 245, 85–96. doi: 10.1007/s00709-010-0144-6 20419461

[B16] Francis C. A. Rutger J. N. Palmer A. F. E. (1969). A rapid method for plant leaf area estimation in maize (*Zea mays* L.). Crop Sci. 9, 537–539. doi: 10.2135/cropsci1969.0011183X000900050005x

[B17] Ghazy N. El-Nahrawy S. (2021). Siderophore production by Bacillus subtilis MF497446 and Pseudomonas koreensis MG209738 and their efficacy in controlling Cephalosporium maydis in maize plant. Arch. Microbiol. 203, 1195–1209. doi: 10.1007/s00203-020-02113-5 33231747 PMC7683328

[B18] Gordon S. A. Weber R. P. (1951). Colorimetric estimation of indoleacetic acid. Plant Physiol. 26, 192–195. doi: 10.1104/pp.26.1.192 16654351 PMC437633

[B19] Hasanuzzaman M. Ahmed N. Saha T. Rahman M. Rahman K. Alam M. M.. (2022a). Exogenous salicylic acid and kinetin modulate reactive oxygen species metabolism and glyoxalase system to confer waterlogging stress tolerance in soybean (*Glycine max* L.). Plant Stress 3, 100057. doi: 10.1016/j.stress.2022.100057

[B20] Hasanuzzaman M. Raihan M. R. H. Nowroz F. Fujita M. (2022b). Insight into the mechanism of salt-induced oxidative stress tolerance in soybean by the application of *Bacillus subtilis*: coordinated actions of osmoregulation, ion homeostasis, antioxidant defense, and methylglyoxal detoxification. Antioxidants 11, 1856. doi: 10.3390/antiox11101856 36290578 PMC9598349

[B21] Heath R. L. Packer L. (1968). Photoperoxidation in isolated chloroplast. I. Kinetics and stoichiometry of fatty acid peroxidation. Arch. Biochem. Biophys. 125, 189–198. doi: 10.1016/0003-9861(68)90654-1 5655425

[B22] Hemeda H. M. Klein B. P. (1990). Effects of naturally occurring antioxidants on peroxidase activity of vegetable extracts. J. Food Sci. 55, 184–185. doi: 10.1111/j.1365-2621.1990.tb06048.x

[B23] Hossain M. A. Nakano Y. Asada K. (1984). Monodehydroascorbate reductase in spinach chloroplasts and its participation in the regeneration of ascorbate for scavenging hydrogen peroxide. Plant Cell Physiol. 25, 385–395. doi: 10.1093/oxfordjournals.pcp.a076726

[B24] Hu Q. (2019). Effects of *Bacillus subtilis* QM3 on germination and antioxidant enzymes activities of wheat seeds under salt stress. Open Access Library J. 6, e5218. doi: 10.4236/oalib.1105218

[B25] Hu Q. Zhao Y. Hu X. Qi J. Suo L. Pan Y.. (2022). Effect of saline land reclamation by constructing the “Raised Field-Shallow Trench” pattern on agroecosystems in Yellow River Delta. Agric. Water Manage. 261, 107345. doi: 10.1016/j.agwat.2021.107345

[B26] Iqbal M. Naveed M. Sanaullah M. Brtnicky M. Hussain M. I. Kucerik J.. (2023). Plant microbe mediated enhancement in growth and yield of canola (*Brassica napus* L.) plant through auxin production and increased nutrient acquisition. J. Soils Sediments 23, 1233–1249. doi: 10.1007/s11368-022-03386-7

[B27] Jahan M. S. Sarker B. C. Ruma A. A. Islam Y. (2023). Effect of salinity stress on growth and yield potential of boro rice. SAARC J. Agric. 21, 1. doi: 10.3329/sja.v21i1.66234

[B28] Ji C. Tian H. Wang X. Song X. Ju R. Li H.. (2022). *Bacillus subtilis* HG-15, a halotolerant rhizoplane bacterium, promotes growth and salinity tolerance in wheat (*Triticum aestivum*). BioMed. Res. Int. 2022, 9506227. doi: 10.1155/2022/9506227 35578723 PMC9107367

[B29] Kanwal R. Maqsood M. F. Shahbaz M. Naz N. Zulfiqar U. Ali M. F.. (2024). Exogenous ascorbic acid as a potent regulator of antioxidants, osmo-protectants, and lipid peroxidation in pea under salt stress. BMC Plant Biol. 24, 247. doi: 10.1186/s12870-024-04947-3 38575856 PMC10996094

[B30] Kapadia C. Patel N. Rana A. Vaidya H. Alfarraj S. Ansari M. J.. (2022). Evaluation of plant growth-promoting and salinity ameliorating potential of halophilic bacteria isolated from saline soil. Front. Plant Sci. 13, 946217. doi: 10.3389/fpls.2022.946217 35909789 PMC9335293

[B31] Khasanov S. Kulmatov R. Li F. van Amstel A. Bartholomeus H. Aslanov I.. (2023). Impact assessment of soil salinity on crop production in Uzbekistan and its global significance. Agric. Ecosys. Environ. 342, 108262. doi: 10.1016/j.agee.2022.108262

[B32] Li H. Qiu Y. Yao T. Ma Y. Zhang H. Yang X. (2020). Effects of PGPR microbial inoculants on the growth and soil properties of *Avena sativa*, *Medicago sativa*, and *Cucumis sativus* seedlings. Soil Tillage Res. 199, 104577. doi: 10.1016/j.still.2020.104577

[B33] Mubeen S. Shahzadi I. Akram W. Saeed W. Yasin N. A. Ahmad A.. (2022). Calcium nanoparticles impregnated with benzenedicarboxylic acid: a new approach to alleviate combined stress of DDT and cadmium in Brassica alboglabra by modulating bioacummulation, antioxidative machinery and osmoregulators. Front. Plant Sci. 13, 825829. doi: 10.3389/fpls.2022.825829 35356123 PMC8959818

[B34] Munns R. (2011). Plant adaptations to salt and water stress: Differences and commonalities. Adv. Bot. Res. 57, 1–32. doi: 10.1016/B978-0-12-387692-8.00001-1

[B35] Nahar K. Hasanuzzaman M. Rahman A. Alam M. M. Mahmud J. A. Suzuki T.. (2016). Polyamines confer salt tolerance in mung bean (*Vigna radiata* L.) by reducing sodium uptake, improving nutrient homeostasis, antioxidant defense, and methylglyoxal detoxification systems. Front. Plant Sci. 7, 1104. doi: 10.3389/fpls.2016.01104 27516763 PMC4964870

[B36] Nakano Y. Asada K. (1981). Hydrogen peroxide is scavenged by ascorbate specific peroxidase in spinach chloroplasts. Plant Cell Physiol. 22, 867–880. doi: 10.1093/oxfordjournals.pcp.a076232

[B37] Principato G. B. Rosi G. Talesa V. Giovanni E. Uotila L. (1987). Purification and characterization of two forms of glyoxalase II from the liver and brain of Wistar rats. Biochim. Biophys. Acta Protein Struct. Mol. Enzymol. 911, 349–355. doi: 10.1016/0167-4838(87)90076-8 3814608

[B38] Puthiyottil P. Akkara Y. (2021). Pre-treatment with *Bacillus subtilis* mitigates drought induced photo-oxidative damages in okra by modulating antioxidant system and photochemical activity. Physiol. Mol. Biol. Plants. 27, 945–957. doi: 10.1007/s12298-021-00982-8 34092946 PMC8140019

[B39] Rahimi S. Talebi M. Baninasab B. Gholami M. Zarei M. Shariatmadari H. (2020). The role of plant growth-promoting rhizobacteria (PGPR) in improving iron acquisition by altering physiological and molecular responses in quince seedlings. Plant Physiol. Biochem. 155, 406–415. doi: 10.1016/j.plaphy.2020.07.045 32814277

[B40] Rajabi D. A. Zahedi M. Piernik A. (2024). Understanding salinity stress responses in sorghum: exploring genotype variability and salt tolerance mechanisms. Front. Plant Sci. 14, 1296286. doi: 10.3389/fpls.2023.1296286 38269142 PMC10806974

[B41] Roman V. J. den Toom L. A. Gamiz C. C. van der Pijl N. Visser R. G. F. van Loo E. N.. (2020). Differential responses to salt stress in ion dynamics, growth and seed yield of European quinoa varieties. Environ. Exp. Bot. 177, 104146. doi: 10.1016/j.envexpbot.2020.104146

[B42] Sharma S. Kumawat K. C. (2022). “Role of rhizospheric microbiome in enhancing plant attributes and soil health for sustainable agriculture,” in Core microbiome: improving crop quality and productivity. Eds. Parray J. A. Shameem N. Abd-Allah E. F. Mir M. Y. (Wiley, Hoboken, New Jersey), 139–162. doi: 10.1002/9781119830795.ch8

[B43] Shultana R. Kee Zuan. A. T. Yusop M. R. Saud H. M. (2020). Characterization of salt-tolerant plant growth-promoting rhizobacteria and the effect on growth and yield of saline-affected rice. PloS One 15, e0238537. doi: 10.1371/journal.pone.0238537 32886707 PMC7473536

[B44] Shultana R. Zuan A. T. K. Yusop M. R. Saud H. M. El-Shehawi A. M. (2021). *Bacillus tequilensis* strain ‘UPMRB9’ improves biochemical attributes and nutrient accumulation in different rice varieties under salinity stress. PloS One 16, e0260869. doi: 10.1371/journal.pone.0260869 34898612 PMC8668098

[B45] Soliman M. H. Abdulmajeed A. M. Alhaithloul H. Alharbi B. M. El-Esawi M. A. Hasanuzzaman M.. (2020). Saponin biopriming positively stimulates antioxidants defense, osmolytes metabolism and ionic status to confer salt stress tolerance in soybean. Acta Physiol. Plant 42, 114. doi: 10.1007/s11738-020-03098-w

[B46] SRDI (2010). “Saline soils of Bangladesh,” in SRMAF Project (Ministry of Agriculture, Bangladesh), 1–60

[B47] Sultana S. Alam S. Karim M. M. (2021). Screening of siderophore-producing salt-tolerant rhizobacteria suitable for supporting plant growth in saline soils with iron limitation. J. Agric. Food Res. 4, 100150. doi: 10.1016/j.jafr.2021.100150

[B48] Sultana S. Paul S. C. Parveen S. Alam S. Rahman N. Jannat B.. (2020). Isolation and identification of salt-tolerant plant-growth-promoting rhizobacteria and their application for rice cultivation under salt stress. Can. J. Microbiol. 66, 144–160. doi: 10.1139/cjm-2019-0323 31714812

[B49] Ullah S. Bano A. Ullah A. Shahid M. A. Khan N. (2022). A comparative study of plant growth promoting rhizobacteria (PGPR) and sowing methods on nutrient availability in wheat and rhizosphere soil under salinity stress. Rhizosphere 23, 100571. doi: 10.1016/j.rhisph.2022.100571

[B50] Wang G. Zhang L. Zhang S. Li B. Li J. Wang X.. (2023). The combined use of a plant growth promoting Bacillus sp. strain and GABA promotes the growth of rice under salt stress by regulating antioxidant enzyme system, enhancing photosynthesis and improving soil enzyme activities. Microbiol. Res. 266, 127225. doi: 10.1016/j.micres.2022.127225 36240664

[B51] Wild R. Ooi L. Srikanth V. Münch G. A. (2012). quick: Convenient and economical method for the reliable determination of methylglyoxal in millimolar concentrations: The N-acetyl-L-cysteine assay. Anal. Bioanal. Chem. 403, 2577–2581. doi: 10.1007/s00216-012-6086-4 22580513

[B52] Woo O. G. Kim H. Kim J. S. Keum H. L. Lee K. C. Sul W. J.. (2020). *Bacillus subtilis* strain GOT9 confers enhanced tolerance to drought and salt stresses in *Arabidopsis thaliana* and *Brassica campestris* . Plant Physiol. Biochem. 148, 359–367. doi: 10.1016/j.plaphy.2020.01.032 32018064

[B53] Yang S.-H. Wang L.-J. Li S.-H. (2007). Ultraviolet-B irradiation-induced freezing tolerance in relation to antioxidant system in winter wheat (*Triticum aestivum* L.) leaves. Environ. Exp. Bot. 60, 300–307. doi: 10.1016/j.envexpbot.2006.12.003

[B54] Yang T. Zhang Y. Guo L. Li D. Liu A. Bilal M.. (2024). Antifreeze polysaccharides from wheat bran: the structural characterization and antifreeze mechanism. Biomacromolecules. doi: 10.1021/acs.biomac.3c00958 38388358

[B55] Zhao Y. Wang H. Song B. Xue P. Zhang W. Peth S.. (2023). Characterizing uncertainty in process-based hydraulic modeling, exemplified in a semiarid Inner Mongolia steppe. Geoderma 440, 116713. doi: 10.1016/j.geoderma.2023.116713

[B56] Zhu A. Wang A. Zhang Y. Dennis E. S. Peacock W. J. Greaves A. I. K. (2020). Early establishment of photosynthesis and auxin biosynthesis plays a key role in early biomass heterosis in *Brassica napus* (Canola) hybrids. Plant Cell Physiol. 61, 1134–1143. doi: 10.1093/pcp/pcaa038 32215572

