# Supplementary material for: Harnessing plant growth-promoting rhizobacteria, Bacillus subtilis and B. aryabhattai to combat salt stress in rice: a study on the regulation of antioxidant defense, ion homeostasis, and photosynthetic parameters
Source: Front Plant Sci. 2024 Jun 13;15:1419764. doi: 10.3389/fpls.2024.1419764 (PMC11208634; doi:10.3389/fpls.2024.1419764)
Supplement: Supplementary file 1 [file DataSheet_1.docx]

**Harnessing Plant Growth-Promoting Rhizobacteria, *Bacillus subtilis* and *B. aryabhattai* to Combat Salt Stress in Rice: A Study on the Regulation of Antioxidant Defense, Ion Homeostasis, and Photosynthetic Parameters**

**Ayesha Siddika^1^, Alfi Anjum Rashid^2^, Shakila Nargis Khan^2^, Amena Khatun^3^, Muhammad Manjurul Karim^2^, PV Vara Prasad^4^* and Mirza Hasanuzzaman^1^***

^1^Department of Agronomy, Faculty of Agriculture, Sher-e-Bangla Agricultural University, Dhaka-1207, Bangladesh

^2^Department of Microbiology, University of Dhaka, Dhaka-1000, Bangladesh

^3^Department of Agriculture, Noakhali Science and Technology University, Noakhali-3814, Bangladesh

^4^Department of Agronomy, Kansas State University, Manhattan, KS, United States

***Correspondence:**

vara@ksu.edu (P.V.V.P.); mhzsauag@yahoo.com (M.H.)

## Supplementary Figures


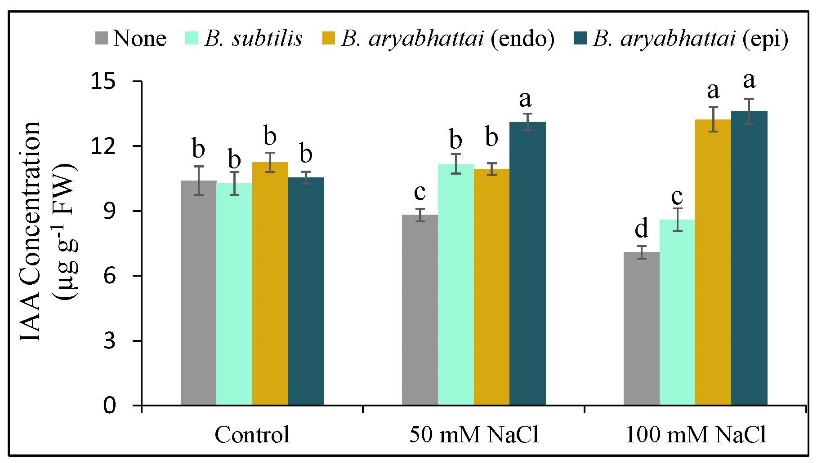


**Supplementary Figure 1.** Changes in the levels of indole-3-acetic acid (IAA) concentration of rice plants under salt stress (50 and 100 mM NaCl) with the absence or presence of three PGPRs (*Bacillus subtilis*, epiphytic *B. aryabhattai*, and endophytic *B. aryabhattai*). Data are presented as mean ± standard deviation of three replications (*n*=3). Distinct letters on the bars show significant differences between treatments at *p* ≤ 0.05 from Tukey’s HSD test.


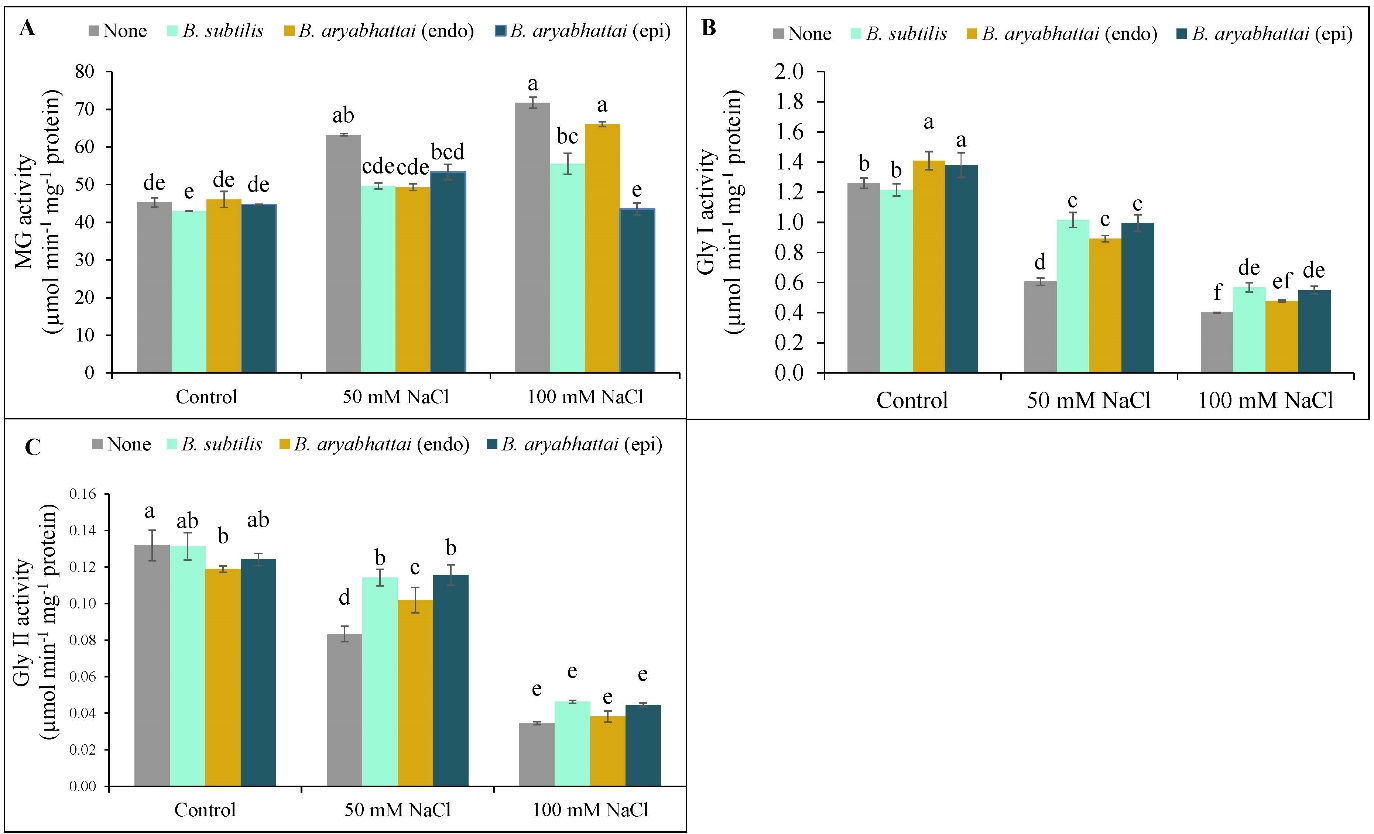


**Supplementary Figure 2.** Variations in MG (A), Gly I (B), and Gly II (C) activities of rice plants under salt stress (50 and 100 mM NaCl) in the absence or presence of three PGPRs (*Bacillus subtilis*, epiphytic *B. aryabhattai*, and endophytic *B. aryabhattai*). Data are presented as mean ± standard deviation of three replications (*n*=3). Distinct letters on the bars show significant differences between treatments at *p* ≤ 0.05 from Tukey’s HSD test.
