# Supplementary figures and images for: Harnessing plant growth-promoting rhizobacteria, Bacillus subtilis and B. aryabhattai to combat salt stress in rice: a study on the regulation of antioxidant defense, ion homeostasis, and photosynthetic parameters
Source: Front Plant Sci. 2024 Jun 13;15:1419764. doi: 10.3389/fpls.2024.1419764 (PMC11208634; doi:10.3389/fpls.2024.1419764)

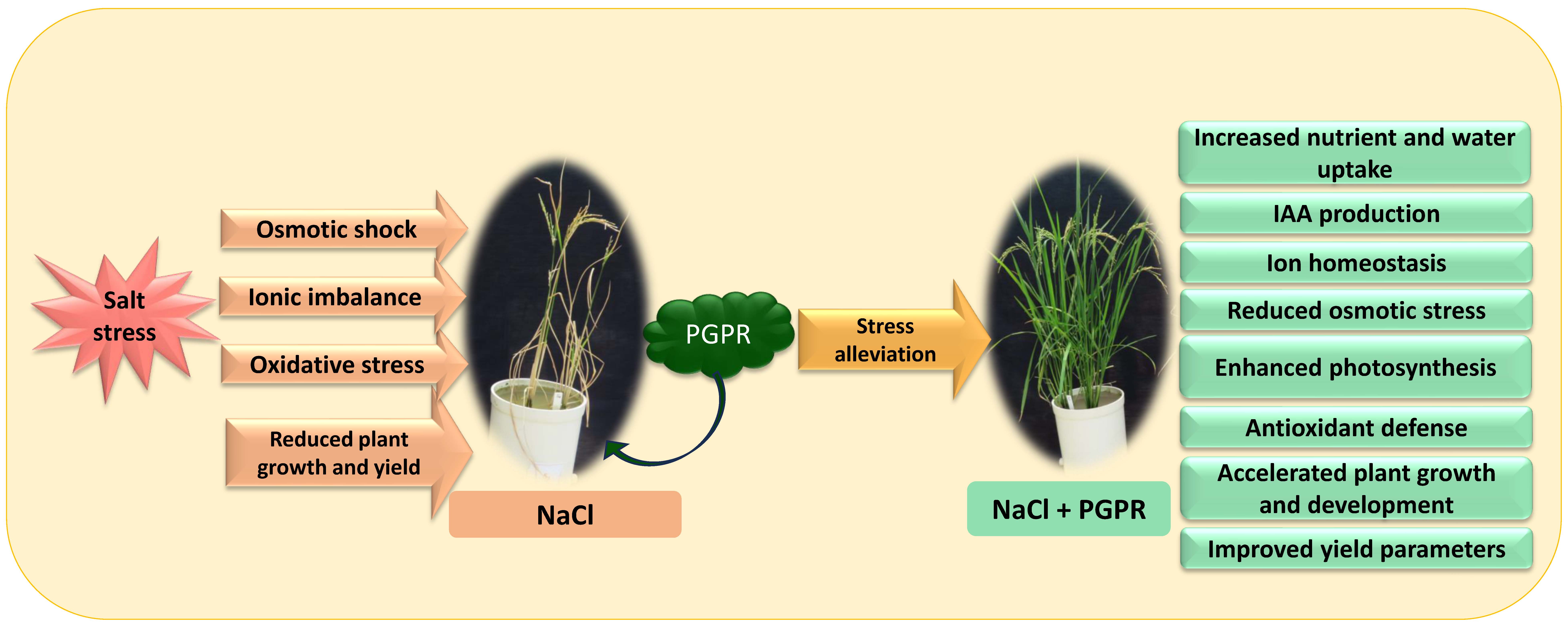

Supplement: Supplementary file 2 [file Image_1.jpeg]
